# Association of Lactoferrin and Toll-like Receptor 2 Genotypes with Mastitis and Milk Components in Vietnamese Holstein Cattle

**DOI:** 10.3390/vetsci9080379

**Published:** 2022-07-25

**Authors:** Lan Doan Pham, Nguyen Van Ba, Le Quang Nam, Phong Vuong Tuan, Duy Ngoc Do

**Affiliations:** 1 Key Laboratory of Animal Cell Technology, National Institute of Animal Sciences, Thuyphuong, Tuliem, Hanoi 100000, Vietnam; nguyenba81@yahoo.com (N.V.B.); chaosongma@yahoo.com (L.Q.N.); 2 Biodiversity Conservation and Tropical Diseases Research Institute, Hanoi 100000, Vietnam; vtphong.spova@vetmed.hokudai.ac.jp; 3 Department of Animal Science and Aquaculture, Dalhousie University, Truro, NS B2N 5E3, Canada

**Keywords:** mastitis, TLR2, LTF, milk composition, Holstein

## Abstract

**Simply Summary:**

Mastitis disease causes huge losses for the dairy industry worldwide. The molecular markers have been shown to be a promising tool for the diagnosis of mastitis infections and the selection of animals with better mastitis resistance. In this study, we surveyed the mastitis incidence in Vietnamese Holstein cows. We also examined the association of polymorphisms in Lactoferrin (LTF) and Toll-like receptor 2 (TLR2) genes with mastitis score and milk component traits. Our results indicated that the interaction between TLR2 genotypes and diseases has a significant impact on the fat and protein percentage of mink. Therefore, TLR2 polymorphism could be used for the selection of cows with better milk compositions.

**Abstract:**

Mastitis is one of the most widespread diseases in dairy cows and causes huge losses for the dairy industry. Molecular markers can be used for the quick diagnosis of mastitis infection, consequently reducing the loss caused by this disease. Lactoferrin (LTF) and Toll-like receptor 2 (TLR2) have been suggested as candidate genes for mastitis; however, their associations with the mastitis incidence and milk components have not been reported in Vietnamese Holstein cows. This study examined the association of TLR2 and LTF polymorphisms with subclinical mastitis and milk components in the Holstein breed raised in Vietnam. Among 192 samples, we identified 44 mastitis-positive samples (22.92%). The mastitis significantly reduced the fat and lactose components in milk (*p* < 0.001) but increased the protein concentration in milk. A total of 94 (49%) and 98 (51%) cows had AA and AB genotypes for the LTF gene, respectively. No significant association was found between the LTF genotypes and the milk component traits or mastitis incidence (*p* > 0.05). The interaction between LTF and mastitis incidence was significantly associated with the protein percentage (*p* = 0.01). A total of 78, 76, and 38 cows had genotypes GG, GT, and TT for the TLR2 gene, respectively. TLR2 genotypes were not significantly associated with mastitis incidence (*p* > 0.05) but were significantly associated with pH value (*p* = 0.03). The interaction between TLR2 and mastitis incidence was significantly associated with the fat (*p* = 0.02) and protein percentage (*p* = 0.04). Further studies are required to confirm the roles of LTF and TFL2 in mastitis in the Holstein breed in Vietnam.

## 1. Introduction

Mastitis is one of the most common and costly diseases in dairy production worldwide. It increases milk production costs and antibiotic residues in milk and decreases milk production and quality [1]. The prevalence of bovine mastitis varies among the countries or even in each region in a country and depends on the production systems. The common pathogens associated with mastitis are *Staphylococcus aureus* (*S. aureus*) and different *Streptococcus* strains [2]. Mastitis is also known to be affected by many factors, including environmental and management factors and genetic factors [3]. The selection for mastitis resistance in cows is possible via genetic or genomic selection programs [4,5,6]. 

Genetic mapping has identified more than 2582 quantitative trait loci (QTL) associated with mastitis resistance or susceptibility on the bovine genome (https://www.animalgenome.org/cgi-bin/QTLdb/BT/index, accessed on 20 March 2022). Many candidate genes for mastitis were also confirmed via a functional genomic approach. Among them, Lactoferrin has been identified as a candidate gene for mastitis resistance in different dairy breeds [7,8,9,10,11]. This gene encodes for the Lactoferrin (LTF) protein, a protein in milk which plays a vital role in the healthy development of newborn mammals [8]. This protein is also an innate resistance factor involved in the prevention of mammary gland infection or mastitis infection. The LTF gene is located on the BTA22 chromosome, consists of 17 exons and 1122 base pairs of the promoter region, and spans about 34.5 kilo base pairs of the genomic DNA. Toll-like receptor 2 (TLR2) is an important gene for mastitis resistance [12,13,14,15,16]. The TLR2 gene locates at the proximal end of BTA17, contains two exons, and encodes 784 amino acids [17]. TLR2 is observed to be strongly expressed during mastitis caused by *S. aureus* [13], which might be reflected in its ability to recognize the peptidoglycan and lipoteichoic acid from *S. aureus* and other gram-positive bacteria [18].

Milk and dairy products are increasingly popular in the Vietnamese diet; consequently, the demand for milk production is also increasing. The Holstein Friesians breed has been imported and has significantly improved milk production in Vietnam. Mastitis is the most common disease of this breed; therefore, it is important to develop effective ways to remove the disease. Using marker-assisted selection or genomic selection is an effective way to control the disease. This study is performed to test the association of TLR2 and LTF genes with mastitis incidence and milk components in the Holstein cattle breed raised in Vietnam.

## 2. Materials and Methods

### 2.1. Resource Population and Phenotypic Records 

The samples were collected according to the standard animal care in Vietnam and as the guideline from the Vietnam National Institute of Animal Science (01/2012). There are no specific laws regarding Animal Welfare in Vietnam so far; therefore, we followed the Vietnamese Law on Animal Health (2015) and the Vietnamese Law on Animal Husbandry (2018). However, these guidelines do not specify details about the use of animals in research. Therefore, we used the guidelines of using animals in research based on EU directive 2010/63 for the best practice during sample collection. 

A total of 192 healthy cows with no incidence of clinical mastitis were randomly selected from the Bavi Cattle and Forage Research Center (Hanoi, Vietnam). All cows were in the middle of lactation and had similar ages (from 2–4 years old). Milk samples were collected in the morning for the California mastitis test (CMT) mastitis diagnosis. The fresh milk from the first quarter of a cow’s mammary gland was used to test with the CMT kits [19]. The California mastitis test is a simple cow-side indicator test for subclinical mastitis that involves the somatic cell count estimation of milk, which allows the DNA in those cells to react with the test reagent, forming a gel [20]. The reaction is scored on a scale of 0 (where the mixture remains unchanged) to 3 (solid gel forms), with a score of 2 or 3 being considered a positive result, following the manufacturer’s recommendation. The 192 milk samples were analyzed for fat (%), lactose (%), protein (%), and pH using the Lactoscan Ultrasonic Milk Analyzer (Milkotronic, Bulgaria) device.

### 2.2. DNA Isolation and Genotyping of Animals 

The methods used to extract DNA have been described before in Pham et al. [21]. Genomic DNA isolation from the ear tissue (around 25 mg) was done using DNeasy Blood & Tissue Kits (Qiagen, Germany) according to the manufactural manual (https://www.qiagen.com/us/products/discovery-and-translational-research/dna-rna-purification/dna-purification/genomic-dna/dneasy-blood-and-tissue-kit/, accessed on 20 April 2020). The quality of genomic DNA was checked by NanoDrop2000 (Thermo Scientific, Waltham, MA, USA) before any further application. The PCR reaction was performed on a 25 μL volume, including 20 ng of genomic DNA, 0.25 μM of each primer, 2.5 μL of 10× PCR buffer (containing 1.5 mM Mg^2+^), 0.2 mM of dNTPs, 1.25U Taq DNA polymerase, and 18.5 μL of double-distilled H_2_O. PCR was carried out on a PCR system PTC-100 with the following procedures: an initial denaturation of 5 min at 95 °C, followed by 35 cycles of 45 s at 94 °C, 30 s at 58 °C, 45 s at 72 °C, and then a 5 min final extension at 72 °C. The amplified DNA (8 μL) was digested at 37 °C with one unit of restriction enzymes (Thermo Scientific, USA) for 4–8 h. The primers and expected product sizes for different genotypes are presented in Table 1. The digests were separated by electrophoresis on 3% agarose gel to extract information about the genotypes. Finally, PCR products of different TLR2 genotypes were sequenced using the ABI 3130 XLcapillary sequencer (Applied Biosystems, Foster City, CA, USA). The obtained sequences of the TLR2 gene were aligned using BioEdit 7.2 software (https://bioedit.software.informer.com/7.2/, accessed on 1 November 2020). 

### 2.3. Statistical Analyses

One-way analysis of variance (ANOVA) and Tukey Honest Significant Differences (TukeyHSD) were used to compare the mastitis CMT scores among TLR2 or LTF genotypes. The significant differences between the mastitis CMT scores among the genotypes were considered if the post-hoc *p* value < 0.05. The differences in the milk components and genotypes between the sub-clinical mastitis and healthy cows were analyzed using a generalized linear model in R. The effects of TLR2, LTF genotypes, and mastitis on the milk components and pH were tested separately using the lmer package [23] in R (version 4.1) and the following linear model: 

y_ijk_ = µ + m_i_ + g_j_ + m_i_×g_j_ + e_k_ where y_ijk_ is the vector for the phenotype, μ is the overall mean of the trait y, m_i_ is the mastitis CMT score, g_j_ is the genotype, m_i_×g_j_ is the interaction between the mastitis and genotypes, and e_k_ is the random error term. The significance of these fixed effects was tested using ANOVA and TukeyHSD, and the *p*-value was considered significant if *p* < 0.05.

## 3. Results

### 3.1. Mastitis Incidence and Milk Components in Vietnamese Cows

Among 192 samples, we identified 44 samples (22.92%) infected with mastitis (Table 2). The fat, protein, and lactose percentages in milk were 4.37 ± 0.11, 2.91 ± 0.04, and 3.90 ± 0.04 in the healthy cows, respectively. The mastitis significantly reduced the fat and lactose components in milk (*p* < 0.001) but increased the protein concentration in milk (from 2.91 ± 0.04 in normal cows to 3.99 ± 0.02 in mastitis cows). There were no significant changes in the pH between the mastitis and the normal cows (*p* = 0.46).

### 3.2. Association between the LTF Genotypes with Mastitis Incidences and Milk Compositions 

**Figure 1:**
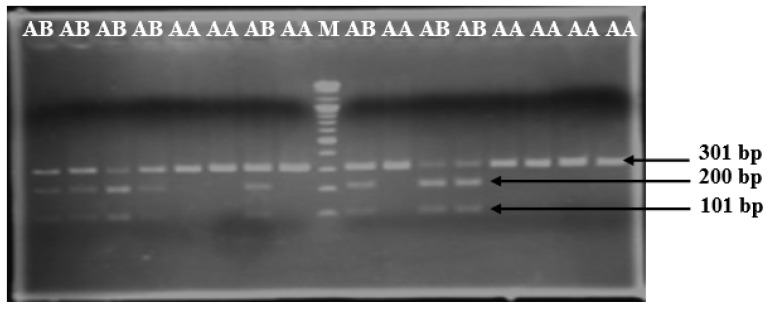
The polymorphism of the Lactoferrin gene. AB and AA indicate the genotypes, and M indicates the leader. Bp: base pairs.

Figure 1 verified the EcoRI-digested LTF gene promoter fragments using the *Eco*RI enzyme in different lanes. The lanes contained unique undigested fragments (301 bp), representing the AA genotype, while the lane with three fragments (301, 201, and 100 bp) showed the AB genotype. The BB genotype was not detected in the current population. 

A total of 94 (49%) and 98 (51%) cows had the genotypes of AA and AB, respectively (Table 3). A higher AA frequency (26) was reported in the mastitis cows compared to the healthy cows (18). However, the TukeyHSD test showed no significant difference between the AA and AB genotypes for the CMT scores (*p* = 0.13). The frequency of A and B was 0.74 and 0.26, respectively. The Hardy–Weinberg test indicated LTF genotypes significantly derived from Hardy–Weinberg equilibrium.

The association analyses showed no significant association between the LTF genotypes and the milk component traits (*p* > 0.05). The interaction between LTF and mastitis incidence was significantly associated with the protein percentage (*p* = 0.01), with a significantly lower protein percentage in healthy cows for both AA and AB genotypes (Table 4).

### 3.3. Association between the Toll-like Receptor 2 Genotypes with Mastitis Incidences and Milk Compositions

**Figure 2:**
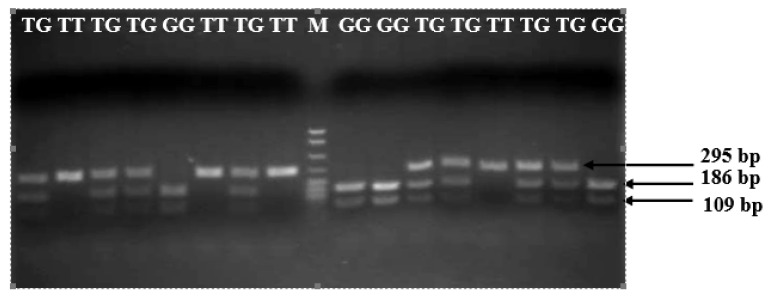
The polymorphism of the TLR2 gene. GG, TG, and TT indicate the genotypes, and M indicates the leader. Bp: base pairs.

**Figure 3:**
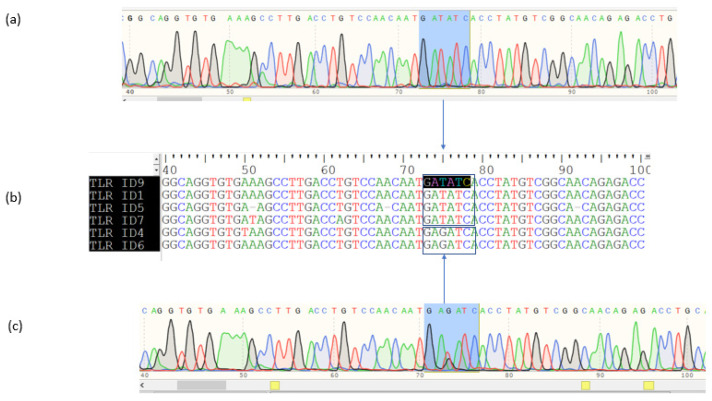
Alignment of the sequence of PCR products for the TLR2 gene. (**a**): The sequences of animals (9, 1, 5, and 7) with TT genotypes; (**b**): The alignment of PCR products for the TLR2 gene for six animals; (**c**): The sequences of animals (4 and 6) with GG or GT genotypes. The blue mark indicated a recognizing sequence of the restriction enzyme, and the error showed the TLR2 alleles (T or G) on this sequence.

Figure 2 showed the three restriction fragments of the *TLR2* gene after digesting with the restriction enzyme. The GG genotypes included two bands, 186 and 109 bp, the GT genotypes included three bands, 295, 186, and 109 bp, and the TT genotypes had only one band with a length of 295 bp (Table 3). The sequences obtained also confirmed the polymorphism in the cutting size of the restriction enzyme. The animals with genotype TT (animals: 9, 1, 5, and 7) contained allele T on the recognized sequence of GA*T*ATC of the restriction enzyme EcoRV (Figure 3a,b), while the animals (4 and 6) that had GG or GT genotypes contained the sequence of GA*G*ATC in the recognized sequence of the restriction enzyme EcoRV (Figure 3b,c). 

A total of 78, 76, and 38 cows have genotypes GG, GT, and TT, respectively. In mastitis cows, the frequency of heterozygous GT genotypes (43%) was higher than that of GG (30%) and TT (27%) genotypes. The frequency of alleles G and T were 0.6 and 0.4, respectively. However, the TukeyHSD test showed no significant difference between the TLR2 genotypes for the CMT scores (*p* > 0.05). The TLR2 genotypes have significantly deviated from the Hardy–Weinberg equilibrium (Table 3).

The association analyses showed a significant association between TLR2 genotypes and pH value (*p* = 0.02) (Table 5). The interaction between TLR2 and mastitis incidence was significantly associated with the fat (*p* =0.02) and protein percentage (*p* = 0.04), with significantly higher values of fat and lower values of protein percentage in healthy cows for both the AA and AB genotypes compared to the values reported for the mastitis cows (Table 5). No significant association between TLR2 and lactose concentration was observed (*p* = 0.68). 

## 4. Discussion

It is well documented that milk components are significantly affected by the breeds, diets, and animal health status [24]. For instance, the concentration of milk fat reported in the current study (4.37%) in healthy cows was higher than the values obtained in Canadian cows (4.04%) [25]. The decrease in the milk fat and lactose percentage caused by mastitis in the current studies was also reported in several other studies [26,27].

The LTF protein is important for the development of calves and other newborn mammals [8]. The polymorphism of the LTF gene has been reported in several breeds [11,22,28]. The absence of the BB genotype for LTF gene in the current study was consistent with the results of Zielak-Steciwko et al. [10], but it was in contrast to the results from Iranian cows [28] and Polish cows [22], which indicated the low frequency of the BB genotype for the LTF gene. The higher A frequency (74%) compared to the B frequency (26%) in the current populations was similar to the percentage of 67.74% for A and that of 32.56% for B in 124 Polish cows [22]. Similarly to Wojdak-Maksymiec [21], the LTF genotypes deviated from the Hardy–Weinberg law in the current population. In this study, we did not observe a significant correlation between the LTF genotypes and the incidence of mastitis, which contrasts with the results reported in Polish cows [22] and Chinese cows [29]. No significant association between LTF genes and mastitis incidence was also observed by Sender et al. [30]. The differences between these studies might be due to the differences in breed compositions, the identification of mastitis, the sample size, and the statistical approaches. Interestingly, we also observed that the LTF polymorphism was significantly associated with the milk protein percentage, which is inconsistent with the results reported by Zielak-Steciwko et al. [10]. The association of LTF genotypes with milk components also depends on the stage of lactations and the positions of the mutations [31]. The LTF genotypes were also reported to be associated with milk yield, milk protein, and fat in Russian cows depending on their lineage [32]. Therefore, a larger sample is required to verify the true association between the LTF gene and milk components in Vietnamese Holstein cows.

The genotypes and allelic frequency of TLR2 depend on the breeds. A higher frequency of the G allele compared to that of the T alleles was also observed in the Simmental population raised in China [14]. TLR2 and its other family members played significant roles in the innate immunity of farm animals [33,34,35]. TLR2 mediates host immune responses in major infectious diseases [36]. Presenting in the cell surface, TLR2 can shape pathogen-specific innate immunity, leading to the development of antigen-specific acquired immunity [36]. The direct association of TLR2 genotypes with mastitis diseases was not consistent in the literature. No association of the TLR2 genotypes with subclinical mastitis was reported in the current study, which has also been confirmed in Norwegian red cattle [37]. In contrast, the significant association of TLR2 with subclinical mastitis has been reported in Chinese Holstein cows [14] and Egyptian Holstein cows [16]. These inconsistencies might be due to the differences in the methods used to score mastitis incidences (CMT versus somatic cell scores), the genotyping methods (PCR-RFLP versus DNA sequencing), and the potential gene due to environmental interaction. Little is known about the association of TLR2 polymorphism with milk production traits. A significant association between TLR2 and fat percentage has been reported in Egyptian cows [16]. The functions of TLR2 in milk components are perhaps rooted in its roles in mastitis and the health of animals. Nevertheless, the results of the current study might be limited by the small sample size used, the CMT method used to characterize the subclinical mastitis incidences, and the genotyping methods. Further studies using a larger sample size and/or DNA sequencing are required to confirm the roles in LTF and TLR2 genotypes in mastitis in the Holstein breed in Vietnam. The association results in the current study also need to be validated using other dairy cattle populations in other regions of Vietnam.

## 5. Conclusions

This study indicated a moderate rate of subclinical mastitis in dairy cows in Vietnam. The mastitis significantly impacted the milk quality. There was no significant association of TLR2 and LTF genotypes with subclinical mastitis incidence in the current study. More samples are required to validate the possibility of using TLR2 and LTF as biomarkers for subclinical mastitis incidence in the Holstein breed in Vietnam.

## Figures and Tables

**Table 1:** PCR primers and expected product sizes for PCR amplicons.

Gene	Location	PCR Primers	PCR Product (Base Pairs)	Enzyme	Expected Digestion Products	References
Lactoferrin	Promoter	F:AACCTACACATGCTGCAATGGAAG R: TGCTTATCGTTCACTGATTGCAGG	301	*Eco*RI	301, 200, and 101	[22]
Toll-like receptor 2	Exon 2	F: CTCTGTCTTGACCCAACT R: ACATAAAGGGACCTGAACC	295	*Eco*RV	295, 186, and 109	[12]

**Table 2:** The milk components changed by mastitis.

Traits	Mastitis (n = 44)	Healthy (n = 148)	*p*
Fat percentage	3.34 ± 0.07	4.37 ± 0.11	<0.001
Protein percentage	3.99 ± 0.02	2.91 ± 0.04	<0.001
Lactose percentage	2.82 ± 0.02	3.90 ± 0.04	<0.001
pH	7.89 ± 0.01	7.94 ± 0.01	0.46

**Table 3:** The genotypic and allelic frequency of Lactoferrin and Toll-like receptor 2 gene.

Gene	Genotypes	Band Size (Base Pairs)	Total	Mastitis	Healthy	Genotype Frequency	Alleles	Alleles Frequency	*p*_HW Test *
*LTF*	AA	301	94	26	68	0.49	A	0.74	
	AB	301, 200 and 101	98	18	80	0.51	B	0.26	<0.001
	BB	200 and 101	0	0	0	0.00			
*TLR2*	GG	186 and 109	78	13	65	0.40	G	0.60	0.016
	GT	295, 186 and 109	76	19	57	0.40	T	0.40	
	TT	295	38	12	26	0.20			

*: *p* values of testing for genotypes derived from the Hardy–Weinberg equilibrium.

**Table 4:** Association between milk components with the Lactoferrin (LTF) genotypes and interactions between Lactoferrin genotypes and mastitis.

Traits	LTF Genotypes	All	*p*	Healthy	Mastitis	*p*
Fat percentage	AA	3.82 ± 0.09	0.69	4.32 ± 0.15	3.33 ± 0.09	0.76
	AB	3.85 ± 0.1		4.3 ± 0.18	3.39 ± 0.08	
Protein percentage	AA	3.43 ± 0.03	0.92	2.85 ± 0.05	4.01 ± 0.03	0.01
	AB	3.5 ± 0.04		3.04 ± 0.07	3.96 ± 0.03	
Lactose percentage	AA	3.35 ± 0.03	0.96	3.87 ± 0.05	2.83 ± 0.03	0.12
	AB	3.38 ± 0.03		3.96 ± 0.05	2.80 ± 0.03	
pH	AA	7.92 ± 0.01	0.41	7.95 ± 0.01	7.89 ± 0.01	0.15
	AB	7.92 ± 0.01		7.94 ± 0.01	7.90± 0.01	

**Table 5:** Association between milk components with the Toll-like receptor 2 (TLR2) genotypes and interactions between Toll-like receptor 2 genotypes and mastitis.

Traits	Genotypes of TLR2	All	*p*_All	Mastitis	Healthy	*p*_Gene × Mastitis
Fat	GG	3.99 ± 0.11	0.53	3.39 ± 0.09	4.58 ± 0.21	0.02
	GT	3.67 ± 0.1		3.4 ± 0.1	3.93 ± 0.17	
	TT	3.91 ± 0.13		3.21 ± 0.15	4.61 ± 0.21	
Protein	GG	3.44 ± 0.04	0.18	3.95 ± 0.03	2.93 ± 0.08	0.04
	GT	3.52 ± 0.04		4 ± 0.04	3.04 ± 0.06	
	TT	3.38 ± 0.05		4.01 ± 0.05	2.75 ± 0.08	
Lactose	GG	3.35 ± 0.03	0.68	2.8 ± 0.03	3.89 ± 0.06	0.23
	GT	3.39 ± 0.03		2.82 ± 0.03	3.96 ± 0.05	
	TT	3.34 ± 0.04		2.86 ± 0.05	3.83 ± 0.07	
pH	GG	7.93 ± 0.01	0.02	7.91 ± 0.01	7.95 ± 0.01	0.45
	GT	7.92 ± 0.00		7.89 ± 0.01	7.95 ± 0.01	
	TT	7.91 ± 0.01		7.89 ± 0.01	7.93 ± 0.01	

*p*_all: Overall *p*_values, *p*_gene x mastitis: *p* values of the testing interaction between genotypes and mastitis.

## Data Availability

The datasets used in this work are available from the corresponding author on academic request.
